# Novel compound heterozygous mutations of LAMA2-limb–girdle muscular dystrophy: A case report and literature review

**DOI:** 10.3389/fneur.2023.1078151

**Published:** 2023-02-13

**Authors:** Duo-Zi Wang, Bing-Hu Li, Qiong Ma, Zhou Yu, Kai Chen, Ying He, Song Tan

**Affiliations:** ^1^Department of Neurology, Sichuan Provincial People's Hospital, University of Electronic Science and Technology of China, Chengdu, China; ^2^Department of Neurology, The First People's Hospital of Liangshan in Yi Autonomous Prefecture, Xichang, China; ^3^Department of Psychosomatic Medicine, Sichuan Provincial People's Hospital, University of Electronic Science and Technology of China, Chengdu, China; ^4^Sichuan Provincial Key Laboratory for Human Disease Gene Study, Chengdu, China; ^5^Chinese Academy of Sciences Sichuan Translational Medicine Research Hospital, Chengdu, Sichuan, China

**Keywords:** limb-girdle muscular dystrophy, LAMA2, merosin, case report, MDC1A, LGMDR23

## Abstract

The laminin α2 (LAMA2) gene pathogenic variants can lead to limb–girdle muscular dystrophy (known as LGMDR23), which is rarely reported and characterized by proximal weakness in the limbs. We present the case of a 52-year-old woman who gradually developed weakness in both lower extremities since the age of 32 years. Magnetic resonance imaging (MRI) brain showed symmetrical sphenoid wings-like white matter demyelination in bilateral lateral ventricles. Electromyography showed quadriceps muscle damage on the bilateral lower extremity. Next-generation sequencing (NGS) found two loci variations in the LAMA2 gene, i.e., c.2749 + 2dup and c.8689C>T. This case highlights the importance of considering LGMDR23 in patients presenting with weakness and white matter demyelination on MRI brain and further expands the gene variants spectrum of LGMDR23.

## 1. Introduction

The laminin α2 (LAMA2) gene localizes on chromosome 6q22-q23, encoding the laminin-α2 chain, which connects with laminin-γ1 and -β1 chains and forms the heterotrimeric laminin-211 protein (1). The LAMA2 pathogenic variants can lead to merosin-deficient congenital muscular dystrophy type 1A (MDC1A) and limb–girdle muscular dystrophy (known as LGMDR23). Together, these two dystrophy types are jointly known as LAMA2-related muscular dystrophy. The former is mainly found in infants and characterized by the early onset of muscle weakness, hypotonia, and joint contractures appearing in infancy or before the first year after birth (2). The latter, while rarely reported, is mainly found in older children and even in adults characterized by proximal weakness of the limbs. In the case report, we reported novel compound heterozygous mutations of the LAMA2 gene in a middle-aged woman with LGMDR23.

## 2. Clinical presentation

A 52-year-old woman presented with dizziness for several days. A few days before the presentation, the patient developed dizziness but denied visual rotation and tinnitus, and denied gait disturbance. Further analysis of medical history revealed that the patient was developmentally normal, with her motor milestones similar to her peers. Since the age of

32 years, the weakness in both lower extremities gradually appeared, but it did not affect her daily life and work. Her son (aged 23 years) and parents had no weakness.

The examination was notable for muscle atrophy of both lower extremities (especially right gluteus maximus) with symmetric proximal weakness (right/left; Medical Research Council [MRC]): deltoids (5/5), biceps (5/5), hip flexors (4/4), hip abductors (4/4), knee flexors (4+/4+), knee extensors (4+/4+). Distal strength of both upper and lower limbs was intact (abductor pollicis brevis [5/5], first dorsal interosseous [5/5], abductor digiti minimi [5/5], finger extensors [5/5], tibialis anterior [5/5], tibialis posterior [5/5], and peroneus longus and brevis [5/5]) and neck flexion/extension was normal. No bulbar muscle weakness or dysarthria were noted. The tone was normal and no fasciculations and clinical myotonia were observed. Deep tendon reflexes were 1+ throughout with flexor plantar responses bilaterally. The remainder of the examination including cranial nerves, sensory modalities, and cerebellar testing was normal. She scored 27 out of 30 on Mini-Mental State Examination (primary school education; ≤20 reflects cognitive impairment), with the following subscores: orientation, 7/10; calculation, 5/5; recall, 6/6; language, 5/5; execute, 3/3; and visuospatial, 1/1.

**Figure 1:**
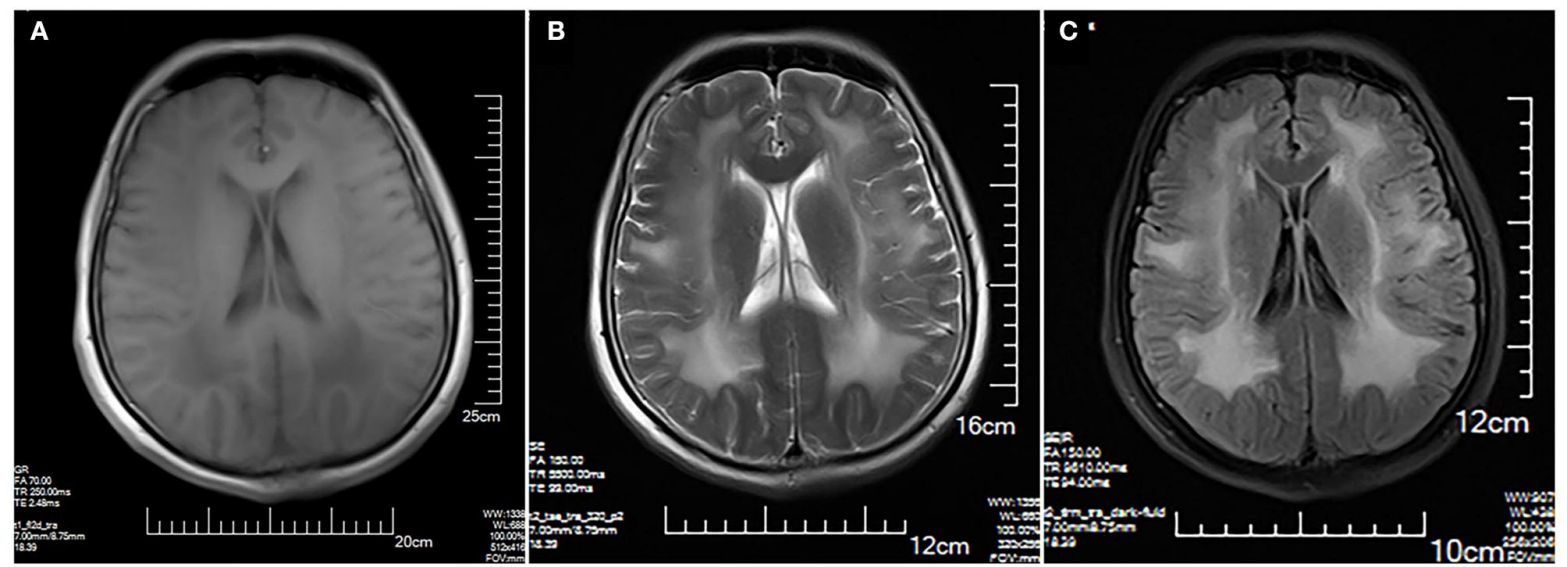
Brain MRI. Symmetrical sphenoid wings-like lesions are shown in bilateral lateral ventricles with slight hypointensity on axial T1 **(A)**; slight hyperintensity on axial T2 **(B)** and FLAIR **(C)**. No obvious abnormality in the sulcus and the gyrus was shown.

**Figure 2:**
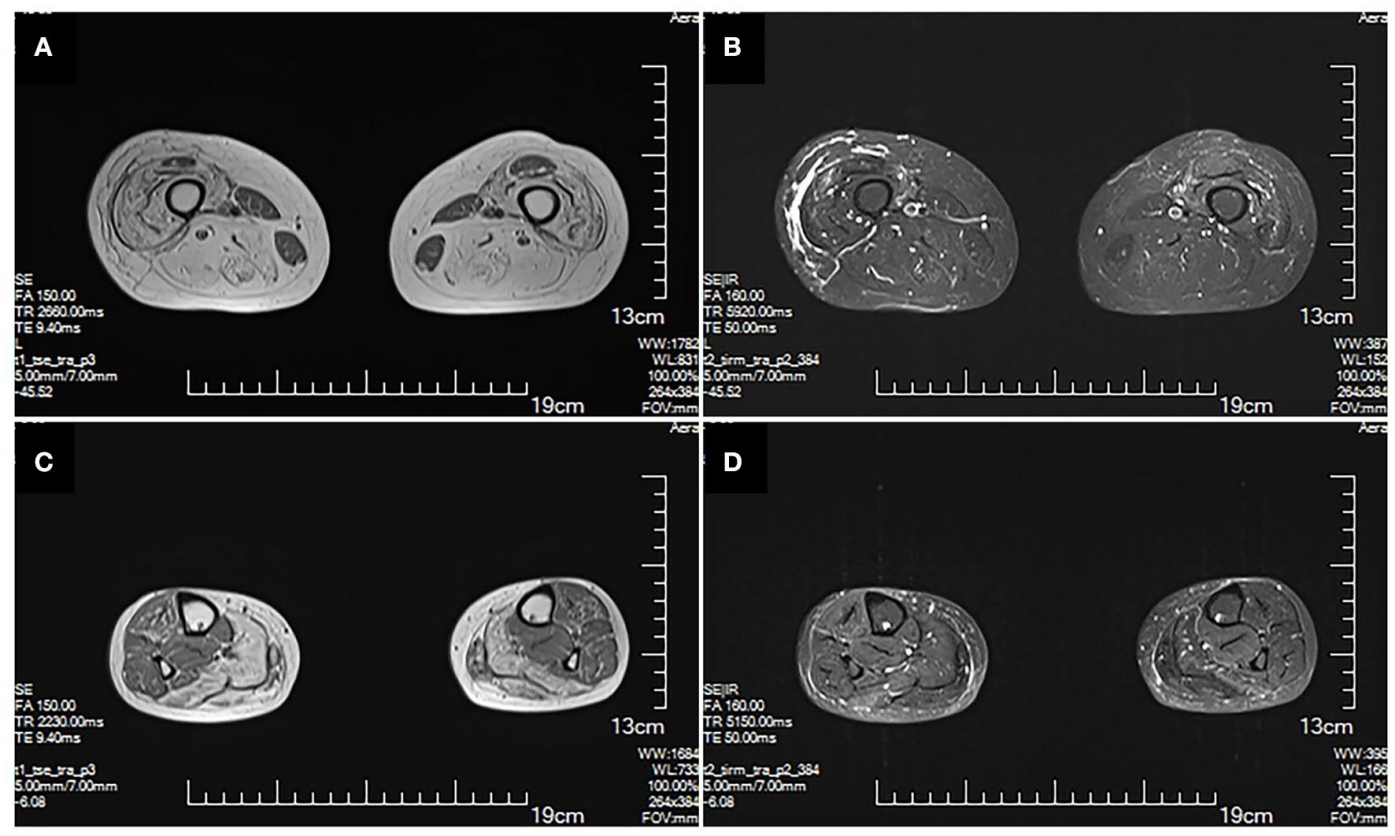
MRI of lower extremities. Muscle MRI T1 **(A)** and T2 **(B)** show diffuse atrophy and fatty degeneration within thigh muscles; the sartorius, the gracilis, and the rectus femoris muscles are selectively preserved. MRI T1 **(C)** and T2 **(D)** of calf muscles show diffuse atrophy and fatty degeneration in the soleus and the lateral head of gastrocnemius muscles. No signs of inflammation and edema are shown.

The MRI brain showed symmetrical sphenoid wings-like lesions in bilateral lateral ventricles with slight hypointensity on T1 and slight hyperintensity on T2 and FLAIR (Figure 1). The MRI muscle showed diffuse atrophy and fatty degeneration in thigh muscles, soleus, and the lateral head of gastrocnemius muscles; the sartorius, the gracilis, and the rectus femoris muscles were selectively preserved (Figure 2).

**Figure 3:**
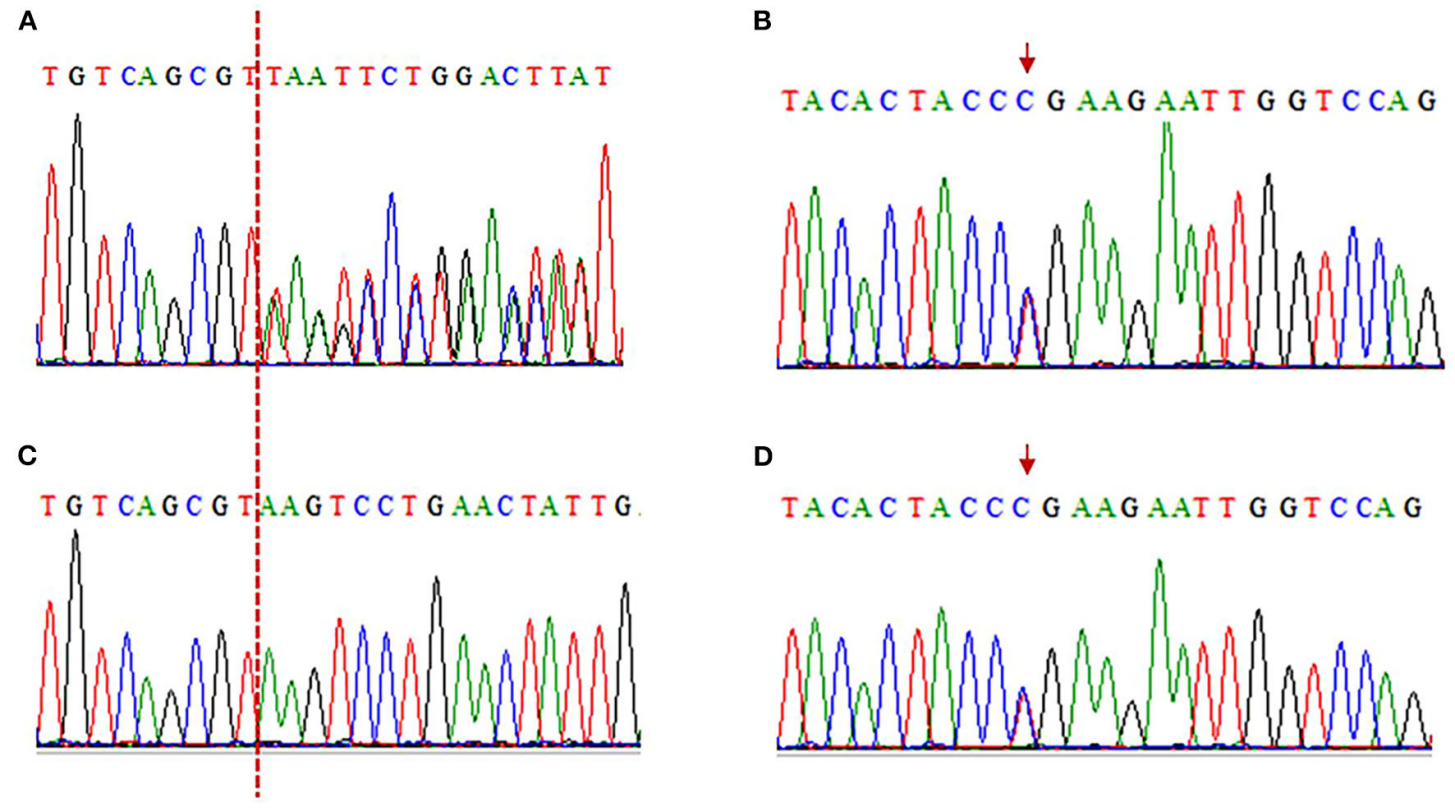
Electropherograms of sanger sequencing results. **(A, C)** The c.2749 + 2dup mutation is shown in the patient, while her son is wild type (A: patient, C: son). The red dotted line indicates the insertion position. c.2749 + 2dup (Reference sequence: TGTCAGCGT(**-**)AAGTCCTGAACTATTG; Mutated sequence: TGTCAGCGT(**T**)AAGTCCTGAACTATT). **(B, D)** The c.8689C>T truncated mutation is in the patient and her son (B: patient, D: son). The red arrows indicate mutation position. c.8689C>T (Reference sequence: TACACTACC**C**GAAGAATTGGTCCAG; Mutated sequence: TACACTACC**T**GAAGAATTGGTCCAG).

Electromyography shows bilateral lower extremity quadriceps muscle damage. The muscle enzymes are slightly elevated (creatine kinase: 445 U/L; normal: 40–200 U/L). Next-generation sequencing (NGS) shows two loci variations in the LAMA2 gene, i.e., c.2749 + 2dup and c.8689C>T (Figure 3). The final diagnosis of LGMDR23 was made.

## 3. Discussion

The possible localizations in this patient with symmetrical lower extremity proximal weakness while lack of sensory involvement include anterior horn cells, motor nerve roots, neuromuscular junction, and muscles. The lack of fasciculations and fatigability makes anterior horn cell conditions (spinal muscular atrophy types 3 and 4) and neuromuscular junction disease (myasthenia gravis) less likely. The chronic condition with adolescence onset makes an inherited myopathy most likely, especially muscular dystrophy. Combined with white matter demyelination, both myotonic dystrophy (MD) (3) and LGMDR23 should be considered. MD is characterized by myotonia, progressive muscle weakness, and atrophy, and it can be genetically classified into MD 1 (caused by an expanded CTG triplet in DMPK on chromosome 19) and MD 2 (caused by the expansion of a CCTG tetramer in CNBP on chromosome 3). The former is characterized by weakness in the distal predominant limbs and grip myotonia, while the latter is characterized by progressive weakness in the proximal and distal limbs and variable mild grip myotonia (4). However, a lack of clinical myotonia makes MD less likely.

LGMDR23 is characterized by proximal weakness in childhood or even adulthood. Patients with LGMDR23 show a mild phenotype with late-onset proximal muscle weakness and delayed motor milestones but achieve independent ambulation (5, 6). As seen in this patient, the weakness rarely affects their life. In terms of the complaint of “dizziness,” we do not believe it is the unique manifestation of LGMDR23 as it disappeared during her follow-up period.

The prevalence of LGMDR23 is unknown; however, it is considered to be very rare. A Chinese study of 130 patients with LAMA2-related muscular dystrophy reported that the frequency of LGMDR23 was 10.8% (14/130) (6). In one study including 128 patients with limb–girdle muscular dystrophy (LGMD), only three patients were due to the mutation of the LAMA2 gene (5). Another study that included 370 patients with LGMD showed that only five patients were due to the mutation of the LAMA2 gene (7). According to previous studies, the age of disease onset ranges from 1 to 56 years. All the reported patients showed mild symptoms and gained independent ambulation and remained ambulatory even in 69-year-old patients. All the reported patients presented with high levels of creatine kinase when diagnosed [from 600 U/L (8) to 2,000 U/L (5)]. A previous study showed the absence of merosin staining in MDC1A, but low levels of residual merosin can be seen in LGMDR23 (5). Unfortunately, immunohistochemistry staining of merosin has not been performed in this case. A few patients showed the involvement of the respiratory and cardiovascular systems. However, most of the patients showed white matter anomalies on brain MRI. In addition, a recent study suggested that diffuse fatty infiltration can be seen in the adductor magnus and in the long head of the biceps femoris selectively.

With respect to the mutations in LGMDR23, the known pathogenic variants included frameshift, nonsense, copy number variations, splicing, missense, and small amino acid deletions, among which the splicing variant was previously assumed to be more common in patients with LGMDR23 (9). In a Chinese LGMDR23 cohort of 14 patients, 23 variants have been reported, among which 8 out of 23 were missense, 7 out of 23 were frameshift, 3 out of 23 were nonsense, 3 out of 23 were copy number variations, 1 out of 23 was splicing, and 1 out of 23 was small amino acid deletions (6). Next-generation sequencing (NGS) found two loci variations in the LAMA2 gene, i.e., c.2749 + 2dup and c.8689C>T in our patient. Both mutations are not reported in the HGMD database (Human Gene Mutation Database). The c.2749 + 2dup is a splicing mutation: a T was inserted between c.2749 + 1 and c.2749 + 2, which is equivalent to the occurrence of T duplication at c.2749 + 2. The c.2749 + 2dup mutation is located in the splicing region, which may cause a splicing change. Aprevious study reported that c.2749 + 2dup might be related to white matter abnormalities (10). In addition, we have made *in silico* analysis by spliceAI, which yielded a score of 0.99 (Table 1), suggesting that this mutation may affect splicing. The c.8689C>T is a truncation mutation, in which CGA mutates into TGA, resulting in the 2,897 arginine being changed to the stop codon and termination of translation at this position. Results of *in silico* analyses by LRT, MutationTaster, Fathmm-MKL, and CADD demonstrated that this mutation may be disease-causing (Table 1). Functionally, this mutation is located in exon 61, encoding concanavalin A-like lectin/glucanase domain|laminin G domain. Thus, this mutation may destruct the interaction between laminins and integrin-α7β1 and αβ-dystroglycan protein complexes, thus disrupting the connection between the basal and muscle cell membranes (11). It was reported that homozygous or compound heterozygous mutations were thought to be pathogenic in theory. Unfortunately, we were unable to perform a sequencing analysis of the patient's parents as they had died. However, as the proband's son carries only one of the two variants, it was confirmed that the proband is a compound heterozygous case. Thus, c.2749 + 2dup and c.8689C>T were considered to be responsible for the proximal weakness in the limbs in this case.

**Table 1:** Results of bioinformatic analyses.

**DNA Variant**	**Protein variant**	**Pathogenicity prediction**							**Splicing prediction**
		**LRT**		**MutationTaste**		**Fathmm-MKL_coding**		**CADD**	**SpliceAI**
		**Score**	**Pred**	**Score**	**Pred**	**Score**	**Pred**		
c.2749 + 2dup	splicing	—	—	—	—	—	—	—	0.99
c.8689C>T	p.R2897X	0	D	1	A	0.847	D	58	0.01
Threshold		D: Deleterious; N: Neutral; U: Unknown		A: Disease causing automatic; D: Disease causing; N: Polymorphism; P: Polymorphism automatic		D: Deleterious; T: Tolerated		>10	>0.5

Score of LRT, MutationTaster, Fathmm-MKL_coding and SpliceAI: 0-1.

CADD, Combined Annotation Dependent Department; threshold: 10, the higher the value, the higher the probability that the mutation site is a harmful mutation.

This case report highlights the importance of considering LGMDR23 in a patient presenting with weakness and white matter demyelination on an MRI of thr brain. It also highlights the importance of comprehensive physical examination in patients with white matter demyelination to find insidious muscle weakness. Moreover, we reported two potentially pathogenic mutations in a compound heterozygous state (c.2749 + 2dup and c.8689C>T) in the LAMA2 gene responsible for LGMD in a Chinese patient, which further expands the gene variants spectrum of LGMDR23. These results can refine prenatal diagnosis, genetic counseling, and treatments of patients with LGMDR23.

## Data availability statement

The datasets presented in this article are not readily available because of ethical and privacy restrictions. Requests to access the datasets should be directed to the corresponding author.

## Ethics statement

The studies involving human participants were reviewed and approved by the Ethical Review Board of Sichuan Provincial People's Hospital. Written informed consent to participate in this study was provided by the patients/participants. Written informed consent was obtained from the patient for the publication of any potentially identifiable images or data included in this article.

## Author contributions

Drafting the manuscript: D-ZW, B-HL, and ST. Obtaining clinical data, critical reviewing of the manuscript, and final approval: All authors.
